# 癌症相关遗传变异的新进展及其在诊断研发中的意义

**DOI:** 10.3779/j.issn.1009-3419.2011.01.17

**Published:** 2011-01-20

**Authors:** William CS CHO, 娟 南

**Affiliations:** 1 Department of Clinical Oncology, Queen Elizabeth Hospital, Hong Kong; 2 天津医科大学总医院，天津市肺癌研究所，天津市肺癌转移与肿瘤微环境重点实验室; 3 香港特别行政区 伊利沙伯医院 临床肿瘤科

**Keywords:** 癌症诊断, 拷贝数目变异（copy number variant, CNV）, 遗传变异, 全基因组关联（genome-wide association, GWA）, 小分子RNA（microRNA, miRNA）, 罕见变异, 单核苷酸多态性（single nucleotide polymorphism, SNP）

**“了解更多癌症相关的遗传变异可能有助于癌症的预防和早期诊断，以及为癌症个体化诊断和治疗方案开辟新途径。”**

遗传变异通过与其它组分或分子相互作用直接或间接地调控基因表达。它们会导致同种异体间的表达差异^[1]^。从理论上讲，了解更多癌症相关的遗传变异可能有助于癌症的预防和早期诊断，以及为个体化癌症诊断和治疗方案开辟新途径。

单核苷酸多态性（single nucleotide polymorphism, SNP）是人类基因组中最常见的遗传变异。公共数据库中储存了逾一千万个SNPs。通过连锁不平衡分析，它们已被证实为检测癌症变异有用的遗传标志物^[2]^。

结构变异也是广泛分布于人类基因组中的遗传变异的重要来源^[3]^。结构变异包括拷贝数目变异（copy number variant, CNV）和中性拷贝变异（copy neutral variation），其中，CNVs包括插入和缺失，而中性拷贝变异包括倒位和易位^[4]^。CNV的特殊位点新生突变率较SNPs频繁100-10, 000余倍^[5]^。CNVs间接影响个体的癌症易感性，如改变抑癌基因或致癌基因的基因数目^[6]^。因此，基因组拷贝数目的改变是许多癌症的特征，且CNV分析有望揭示参与癌变的基因^[7]^。

## 癌症的全基因组关联研究

全基因组关联（genome-wide association, GWA）研究在基因-癌症关联的发现中引起了划时代的变革。GWA研究的最终目标是形成一种整合遗传、环境、自身危险因素的综合危险预测模型，以利疾病的诊断、预防和治疗^[8]^。在过去的几年里，已鉴定出许多常见癌症的新型遗传变异^[9]^。本文将重点介绍那些有助于新型诊断和治疗策略的发展之遗传变异。

## 前列腺癌

在欧洲人群中，染色体8q24上存在rs1447295和rs6983267可导致前列腺癌^[10]^。染色体8q24上的易感性变异在欧洲人群中相对罕见（2%-4%），而在非裔美籍人群中则较为常见（~42%），这易感性变异与诊断时患者年纪较小有关^[11]^。在选定个体中已鉴定出许多可预测高风险的易感性基因，包括*HNF1B*、*MSMB*、*CTBP2*、*JAZF1*、*CPNE3*、*IL16*和*CDH13*^[12]^。一个研究小组显示有两种遗传变异（Xp11.22上的rs5945572和2p15上的rs721048）与前列腺癌的侵袭性密切相关^[13]^。他们还报道位居5种遗传变异（3q21.3上的rs10934853[A]、8q24.21上的rs16902094[G]和rs445114[T]、11q13上的rs11228565[A]和19q13.2上的rs8102476[C]）的危险因素分布首1.3%的欧洲人群罹患前列腺癌的风险增高2.5倍^[14]^。

## 乳腺癌

*FGFR2*基因内含子2的SNP rs1219648已被证实与个别绝经后乳腺癌的风险相关^[15]^。有4个遗传变异（2q35上的rs13387042[A]、5p12上邻近*MRPS30*的rs4415084和rs10941679、以及16q12上邻近*TNRC9*的rs3803662[T]）仅与雌激素受体阳性肿瘤的风险相关^[16, 17]^。其它乳腺癌易感性遗传变异包括1p11.2上邻近*NOTCH2*和*FCGR1B*的rs11249433、3p24上包括*SLC4A7*和*NEK10*的rs4973768、6q25.1上位于*ESR1*上游的rs2046210、8q24上的rs1562430、14q24.1上驻于*RAD51L1*的rs999737以及17q23.2上包括*COX11*的rs6504950^[18-21]^。

## 结直肠癌与胃癌

GWA研究已鉴定了许多结直肠癌易感性变异（8q23.3上标识*EIF3H*的rs16892766、8q24上的rs6983267和rs7014346、10p14上的rs10795668、11q23上的rs3802842、18q21上的rs4939827）^[22-24]^。这些发现拓宽了我们对常见遗传变异在结直肠癌病因学中的作用的理解。此外，一项GWA研究鉴定了一种前列腺干细胞抗原遗传变异（rs2976392），它可能参与调节胃上皮细胞增殖，并影响弥漫型胃癌的易感性^[25]^。

## 白血病

3个急性淋巴细胞白血病的风险变异（7p12.2上邻近*IKZF1*的rs4132601、10q21.2上邻近*ARID5B*的rs7089424和14q11.2上邻近*CEBPE*的rs2239633）可能参与祖B-细胞的转录调节和分化^[26]^。此外，已鉴定出12个慢性淋巴细胞白血病的风险变异（2q13上的rs17483466、2q37.1邻近*SP140*的rs13397985、2q37.3上邻近*FARP2*的rs757978、6p25.3上邻近*IRF4*的rs872071、8q24.21上的rs2456449、11q24.1上的rs735665、15q21.3上的rs7169431、15q23上的rs7176508、15q25.2上邻近*CPEB1*的rs783540、16q24.1上的rs305061、18q21.1上的rs1036935和19q13.32上邻近*PRKD2*的rs11083846），它们可能为这血癌的病因提供新的见解^[27, 28]^。

## 黑色素瘤与基底細胞癌

有2个遗传变异（第20号染色体上的rs910873和rs1885120）被鉴定为黑色素瘤风险位点，在早期发病的病例中具有强烈相关性^[29]^，而在欧洲人群中有3个遗传变异（9p21上邻近*MTAP*和*CDKN2A*的rs7023329、11q14-q21上包含*TYR*的rs1393350和16q24上包含*MC1R*的rs258322）与黑色素瘤风险具有独立的相关性^[30]^。有些遗传变异（1q42上邻近*RHOU*的rs801114、1p36上包括*PADI4*、*PADI6*、*RCC2*和*ARHGFF10L*的rs7538876、9p21上邻近*CDKN2A*和*CDKN2B*的rs2151280[C]、7q32上邻近印记基因*KLF14*的rs157935[T]以及*KRT5*中编码G138E替代的rs11170164）与基底細胞癌易感性相关，但与黑色素瘤或着色性状无关。确凿的证据表明*TERT-CLPTM1L*位点的rs401681[C]与基底細胞癌易感性相关，但对黑色素瘤有防护作用^[31, 32]^。

**“有研究假设*EGFR*拷贝数增多与肺癌强侵袭性和低分化相关”**

## 肺癌

GWA研究也鉴定了一些疾病标记物（5p15.33上包括*CLPTM1L*和*TERT*的rs401681、rs402710和rs2736100、6p21.33上匹配*BAT3-MSH5*的rs3117582、15q25.1上包括*PSMA4*、*CHRNA3*和*CHRNA5*的rs1051730和rs8034191）与肺癌风险显著相关，表明这些遗传变异可能在肺癌发病因素中发挥作用^[33-35]^。有临床研究显示，*EGFR*基因拷贝数目越多，采用吉非替尼治疗的效果就越好^[36]^，还有研究假设，*EGFR*拷贝数目增多与肺癌强侵袭性和低分化相关^[37-39]^。

## 神经胶质瘤与神经母细胞瘤

一项神经母细胞瘤的GWA研究发现了两个遗传变异（2q35上*BARD1*位点内的rs3768716和rs6435862），它们导致临床密切相关亚型人神经母细胞瘤侵袭的发生^[40]^。神经胶质瘤的研究发现，有两个遗传变异（9p21上邻近*CDKN2B*的rs1412829和20q13.3上内含*RTEL1*的rs6010620）与高恶性度神经胶质瘤的易感性相关^[41]^。

## 胰腺癌

两项GWA研究已鉴定了一些与胰腺癌相关的遗传变异（1q32.1上匹配*NR5A2*的rs3790844、5p15.33上匹配*CLPTM1L-TERT*的rs401681、9q34上的rs505922、13q22.1上的rs9543325和rs9564966）^[42, 43]^。SNP rs505922与*ABO*血型基因第一内含子匹配，表明与A型或B型相比，O型血患者罹患胰腺癌的风险较低^[42]^。

## 鼻咽癌

一项鼻咽癌的GWA研究鉴定了3个易感性变异（3q26上邻近*MDS1–EVI1*的rs6774494、9p21上邻近*CDKN2A–CDKN2B*的rs1412829、13q12上邻近*TNFRSF19*的rs9510787）。这些发现通过强调鼻咽癌涉及*TNFRSF19*和*MDS1–EVI1*相关通路以及HLA分子，为其发病机制提供了新见解^[44]^。

## MicroRNA靶点的SNPs

作为主要的基因调节因子，microRNAs（miRNAs）是细胞通路的重要调控因子，它们在肿瘤形成中发挥关键作用^[45]^。有些miRNAs被证实为致癌基因、抑癌基因或肿瘤干细胞和转移的调控因子^[46]^。许多位点与基因组的非编码区结合，而miRNA位点或靶点的SNPs或可作为癌症风险的低外显率修饰因子^[47]^。因此，研究miRNA靶点SNPs的功能作用有助于诊断的研发^[48]^。在过去的几年里，基于SNPs的miRNA研究已鉴定了许多与各种癌症相关的遗传变异^[49]^，本文将重点关注其中一部分。

在中度吸烟者中*KRAS* 3'非翻译区的*let-7*互补位点LCS6的SNP与非小细胞肺癌风险的增加显著相关^[50]^。在绝经前的女性中，*ESR1*中*miR*‑*453*靶点内的另一变异（rs2747648）与家族性乳腺癌风险的增强在临床上具有相关性^[51]^。*MiR-196a2*位点的变异（rs11614913, C/T）与肺癌、乳腺癌、食道癌、胃癌和头颈癌相关^[52]^。

**“大部分已鉴定的遗传变异只与相对少的癌症风险有关，且仅能解释很小比例的家族性聚集。”**

## 罕见变异

尽管最近公布了许多GWA研究的重要成果，但是癌症的许多遗传风险仍未明瞭。大部分已鉴定的遗传变异只与相对少的癌症风险有关，且仅能解释很小比例的家族性聚集^[53]^。这意味着遗传风险的盲区。许多未知的遗传控制是由现有GWA研究无法筛选的罕见变异造成的，这些罕见变异对疾病风险的影响相对较大^[54]^。全力鉴定罕见变异导致候选基因的系统筛查。新一代测序技术的出现可大幅提高生成数据的速度和容量，免除克隆偏见以及毛细管测序时标本准备的困难^[55]^。1000基因组项目亦鉴定了许多低频等位基因的遗传变异^[101]^。

## 癌症相关遗传变异在诊断发展中的意义

在过去的几年中，GWA研究被证实在鉴定各种癌症的遗传变异中是有效的。无论类型（SNP或CNV）和频率（常见或罕见变异），数以百计或甚至千计的基因变异与癌症风险有关。这些变异可能为诊断、患者分层和治疗或预后分类提供有用的标记物。此外，汇编遗传变异有助于将疾病中调控细胞活性的生物通路联系起来。常见通路的遗传异质性的鉴定，或有利于根据致病基因位点的生物化学靶点设计新的诊断试验，并制定新的治疗策略以控制癌症的肆虐^[56]^。

**“若结合家族史信息，遗传变异将更具有临床实用性……”**

有些公司已开始为个体化风险预测提供SNP基因分型。然而，已知的SNP风险等位基因的预测效能仍然有限，有待发现更多的变异来提高它们在癌症筛查中的意义。若结合家族史信息，遗传变异将更具有临床实用性，如决定哪些患者应接受结肠镜检查或乳腺癌的MRI筛查^[57]^。

## 未来挑战

在GWA研究的时代，预计最近GWA研究的趋势将持续。尽管已鉴定出了许多癌症的新型遗传变异，但鉴定有实际功能的遗传变异的工作仍待进行。由于鉴定罕见SNPs时统计学效能显著降低，因此有待增加样本数量来检测罕见变异。恰当的研究设计在决定GWA研究的成败中亦起关键作用。芯片需包含更多SNPs的罕见变异，并应采用严格的对照组来提高基因分型的准确性。也需发展整合分析CNVs和GWA的研究方法，包括GWA序列设计的创新和SNPs与常见拷贝数目多态性间连锁不平衡关系的使用。为了获得更有意义的荟萃分析结果，建议采用更具有一致性的实验设计和统计方法。我们在理解遗传变异在癌症治疗中的作用方面仍处于早期阶段。接下来的研究趋势是整合各种有用的DNA和RNA信息（编码与非编码转录）的生物组学技术和鉴定癌症诊断或治疗进展的遗传变异的数据库^[58, 59]^。

## Financial & competing interests disclosure

The author has no relevant affiliations or financial involvement with any organization or entity with a financial interest in or financial conflict with the subject matter or materials discussed in the manuscript. This includes employment, consultancies, honoraria, stock ownership or options, expert testimony, grants or patients received or pending, or royalties. No writing assistance was utilized in the production of this manuscript.
